# Obstructive Sleep Apnea and Cardiovascular Diseases: Sad Realities and Untold Truths regarding Care of Patients in 2022

**DOI:** 10.1155/2022/6006127

**Published:** 2022-08-11

**Authors:** Satya Preetham Gunta, Roopesh Sai Jakulla, Aamer Ubaid, Kareem Mohamed, Abid Bhat, Angel López-Candales, Nicholas Norgard

**Affiliations:** ^1^Department of Internal Medicine, University of Missouri-Kansas City, Kansas City, Missouri, USA; ^2^Department of Sleep Medicine, University of Missouri-Kansas City, Kansas City, Missouri, USA; ^3^Department of Cardiovascular Diseases, University of Missouri-Kansas City, Kansas City, Missouri, USA

## Abstract

Obstructive sleep apnea (OSA) is one of the most common and serious sleep-related breathing disorders with a high prevalence among patients with cardiovascular (CV) diseases. Despite its widespread presence, OSA remains severely undiagnosed and untreated. CV mortality and morbidity are significantly increased in the presence of OSA as it is associated with an increased risk of resistant hypertension, heart failure, arrhythmias, and coronary artery disease. Evaluation and treatment of OSA should focus on recognizing patients at risk of developing OSA. The use of screening questionnaires should be routine, but a formal polysomnography sleep study is fundamental in establishing and classifying OSA. Recognition of OSA patients will allow for the institution of appropriate therapy that should alleviate OSA-related symptoms with the intent of decreasing adverse CV risk. In this review, we focus on the impact OSA has on CV disease and evaluate contemporary OSA treatments. Our goal is to heighten awareness among CV practitioners.

## 1. Introduction

Obstructive sleep apnea (OSA) is one of the most common and serious sleep-related breathing disorders. It is characterized by repetitive upper airway collapse during sleep, which can lead to sleep fragmentation, oxygen desaturation, autonomic dysfunction, and excessive daytime sleepiness. Besides reducing a person's quality of life, the acute and chronic physiologic stresses from repetitive upper airway obstruction can lead to increased cardiovascular (CV) morbidity and mortality. In fact, OSA is highly prevalent in patients with CV disease, estimated to occur in more than 40% of such patients. Despite its widespread presence, OSA remains severely undiagnosed and untreated, which elevates the risk of OSA-related long-term consequences [1].

Evaluation and treatment of OSA should focus on recognizing patients at risk of developing OSA. Recognition of these patients not only will allow diagnosis but most importantly institute appropriate therapy that should alleviate OSA-related symptoms with the intent of decreasing adverse CV risk. In this review, we focus on the impact OSA has on CV disease and evaluate contemporary OSA treatments. Our goal is to heighten awareness among CV practitioners.

## 2. Diagnosis

As a clinical entity, OSA is widely underdiagnosed. Not only can many confounding variables limit both patient and physician awareness, but also logistical and financial barriers can significantly hinder adequate screening. Screening for OSA should be routine in patients with CVD given the high prevalence and comorbidity of OSA among such patients. Risk factors for OSA are listed in Box 1. There are several widely recognized screening questionnaires that are reasonably easy to administer: the Berlin Questionnaire, Stop, STOP-BANG, Epworth Sleepiness Scale (ESS), and 4-Variable screening tool (4-V). These questionnaires include the assessment of OSA symptoms and risk factors. The STOP-BANG is the most sensitive screening tool and one of the easiest to administer quickly in clinic [2]. However, these OSA screening questionnaires have limited specificity and appear to have poorer diagnostic accuracy in certain groups such as in women and in patients with underlying CVD [3]. Therefore, OSA screening questionnaires are not recommended as a replacement for formal sleep testing to diagnose or exclude obstructive sleep apnea [4].

The gold standard test for the diagnosis of sleep apnea is the attended in-hospital polysomnography (PSG) as it is the best method of detecting apneas and hypopneas, staging sleep, quantifying sleep fragmentation, and identifying other sleep-related phenomena [5]. An OSA diagnosis requires the presence of OSA symptoms (nocturnal breathing disturbances or daytime sleepiness) in combination with an apnea-hypopnea index (AHI) greater than 5 [4]. OSA is empirically categorized based on AHI: mild OSA 5/h to <15/h, moderate OSA 15/h to 30/h, and severe OSA > 30/h [4]. CVD incidence and morbidity are more strongly associated with severe OSA as well as a high overnight hypoxia burden [6–9].

Financial constraints, long wait times, and patient logistical issues hinder the use of PSG [10, 11]. As a result of these limitations, home sleep apnea testing (HSAT) has become used more commonly due to their lower cost and lessened technically complexity [12–14]. An HSAT cannot calculate an AHI but rather provides a respiratory event index (REI), which is the sum of apneas and hypopneas divided by the estimated sleep time. HSAT lacks the sensitivity of in-laboratory PSG. While HSAT REI-based OSA diagnosis and categorization use the same scale as the AHI cutoffs, the REI has the potential to underestimate apnea or hypopnea events per hour of sleep, which may lead to a hazardous misclassification of OSA and a denial of a beneficial therapy. HSAT methods are not recommended in patients with significant cardiopulmonary disease [4]. Additionally, if used, an HSAT providing a negative, inconclusive, or technically inadequate result requires a confirmatory in-laboratory PSG.

## 3. Clinical Comorbidities Associated with OSA

OSA has been associated with several CV complications including hypertension, heart failure (HF), coronary artery disease, arrhythmias, and CV mortality. Repeated nocturnal apneic episodes initiate an array of pathophysiological mechanisms, which may act to promote cardiac and vascular disease. OSA-related hypoxia and hypercapnia trigger chemoreflex-mediated increases in sympathetic activity [15]. The consequent sympathetic-mediated vasoconstriction can result in blood pressure surges and extreme hemodynamic stress. A meta-analysis also showed significantly higher levels of angiotensin II and aldosterone levels among patients with OSA partially because of increased RAAS activity [16]. Intermittent hypoxemia and sleep deprivation are important activators of proinflammatory pathways leading to a systemic inflammatory state. OSA-related sympathetic hyperactivation, pressure surges, and systemic inflammation may all contribute to the development of endothelial dysfunction and a prothrombotic state. Additionally, changes in intrathoracic pressure can alter cardiac transmural gradients and disrupt ventricular function leading to increased wall stress and impaired diastolic function.

### 3.1. Hypertension

Hypertension and OSA often coexist as an estimated 50% of OSA patients are hypertensive and 30% of hypertensive patients also have OSA [17–19]. OSA can often precede and predict the development of hypertension. In fact, the risk of developing hypertension is increased 2- to 3-fold in patients with untreated OSA [20]. A diagnosis of moderate-severe OSA has been implicated as an important independent risk factor for resistant hypertension [21]. Among patients with resistant hypertension, ~80% may have OSA [22, 23]. An increase in AHI was found to be linearly matched by an increase in 24-hour blood pressure [24, 25]. However, OSA may have its biggest impact on the blunting of the normal nocturnal fall in blood pressure [26]. Attenuation of nocturnal blood pressure dipping (nondipping) has been linked with an increase in all-cause mortality [27].

### 3.2. Heart Failure

OSA is prevalent in HF and associated with adverse HF outcomes [28]. The Sleep-Disordered Breathing in Heart Failure (SchlaHF) Registry identified the prevalence of moderate-severe sleep-disordered breathing to be 49% in men and 36% in women with stable, symptomatic HF with reduced ejection fraction [29]. OSA has also been estimated to be present in >50% of HF patients with preserved ejection fraction [30]. Nevertheless, it is likely that descriptions of OSA frequency are underestimating the prevalence of OSA in this high-risk patient population because important indicators of OSA such as daytime sleepiness are masked by excessive sympathetic activation and rarely reported by HF patients. Interestingly, HF has also been seen to have a causal association with sleep-disordered breathing. It is postulated that the “nocturnal rostral fluid shift” in HF patients can lead to either pulmonary congestion or fluid accumulation in the neck. This can lead to central or obstructive sleep apnea, respectively. Use of diuretics helping with the severity of sleep apnea aids this theory [31].

Sleep apnea can lead to HF disease advancement and decompensation leading to symptom progression, hospitalization, and mortality [28]. AHI is not the best measure of disease burden in HF patients. Rather, the best prognostic predictor is the hypoxemic burden (time with oxygen saturation less than 90%) [6]. In fact, for each hypoxemic hour, the risk of death is increased by 16.1% [6].

### 3.3. Arrhythmias

Up to 50% of OSA patients experience nocturnal arrhythmias [32–34]. A diagnosis of sleep apnea leads to a 2- to 4-fold increased risk of nocturnal arrhythmias [35]. OSA-related arrhythmias tend to occur after respiratory events and can manifest across a spectrum of cardiac rhythm disturbances including bradyarrhythmias, supraventricular and ventricular tachyarrhythmias, and sudden cardiac arrest [36].

Atrial fibrillation has been strongly associated with OSA with an estimated prevalence of 21% to 74% [37, 38]. OSA and atrial fibrillation share many common risk factors, such as increased age, obesity, and hypertension, which may act together to increase risk [39]. OSA has been shown to independently induce cardiac structural changes such as left atrial enlargement that can predispose a patient to atrial fibrillation [40]. The magnitude of nocturnal oxygen desaturation has been found to be a strong predictor of incident atrial fibrillation [41]. An investigation of night-to-night variability in OSA severity found that nights with the highest severity had an increased risk of having atrial fibrillation during the same day [42]. The presence of OSA has also been shown to attenuate the efficacy of catheter-based and pharmacological antiarrhythmic treatments in AF patients [43, 44].

Bradyarrhythmic events are also common in OSA patients, and their incidence is related to OSA severity [45]. Polysomnographic studies have shown a high prevalence (59%) of sleep apnea syndrome in patients with pacemakers [46]. OSA episodes are associated with parasympathetic stimulation during early phases of apnea, which can elicit a discernible bradycardia.

Patients with OSA are at a higher risk of ventricular arrhythmias having been reported in up to 66% of patients with sleep apnea [33]. While premature ventricular contractions are most common, severe OSA has been reported to be associated with an increased risk of sudden cardiac death, which is usually considered to be due to a ventricular arrhythmia [47]. Coexisting HF and sleep apnea increase the risk of developing malignant ventricular arrhythmias [48]. Furthermore, in HF patients with an implantable cardioverter-defibrillator, OSA was found to be an independent predictor of life-threatening ventricular arrhythmias, which occur more often during sleep, with the greatest frequency occurring during apneic periods [49–51].

### 3.4. Coronary Artery Disease

OSA is associated with increased risk of coronary disease and vascular events. A large observational cohort study of more than 1400 patients showed that the presence of OSA increased the risk 2-fold for the development of myocardial infarction, coronary revascularization events, or CV death, independent of other CV risk factors [52]. Likewise, epidemiological studies have shown that OSA is present in 38% to 65% of patients with coronary artery disease and about 50% of patients requiring percutaneous coronary intervention (PCI) [53, 54]. Regarding patients requiring PCI, OSA appears to increase the risk of cardiac death, nonfatal MI, and coronary revascularization following the procedure [55, 56].

In a meta-analysis, OSA has been found to independently increase the risk of subclinical CAD as measured by coronary artery calcification score [57]. Likewise, OSA was found to be an independent predictor of atherosclerotic plaque progression and vulnerability [58]. In a smaller study, noncalcified plaque burden significantly correlated with AHI while the Coronary Artery Calcium (CAC) score did not. Severe nocturnal hypoxemia can provoke electrocardiographic signs of ischemia (ST depression), and the traditional time window for the onset of a myocardial infarction is shifted to more likely occur during the nighttime in patients with OSA [59].

### 3.5. Diabetes Mellitus

While OSA and type 2 DM have common risk factors like obesity and aging, evidence to suggest a causative association between the two has been growing. Laboratory experiments in healthy human subjects have shown that inadequate sleep and intermittent hypoxemia can impair glucose metabolism and insulin sensitivity [60–62]. Multiple longitudinal cohort studies, after adjusting for confounders, have also demonstrated a significant association between OSA and incident diabetes mellitus [63]. A recent systematic review and meta-analysis suggested CPAP treatment in patients with OSA and insulin resistance could stop and reverse metabolic dysfunction [64]. But other studies do not show any favorable effect of CPAP on metabolic dysfunction [63].

### 3.6. Dyslipidemia

Studies in mice have demonstrated the effect of intermittent hypoxemia and tissue hypoxia on increased lipolysis and triglyceride levels [65, 66]. In the Sleep Heart Health Study which included close to 4500 patients below the age of 65, it was seen that serum total cholesterol and triglyceride levels correlate with the severity of OSA [67]. Numerous observational studies in OSA patients have shown variable results from no effect on lipid levels to elevated total cholesterol, triglycerides, and reduced HDL levels. The studies on the effect of CPAP on lipid levels are limited in evidence with mixed results [68]. Furthermore, the relationship between OSA and metabolic dysregulation has been extended to hepatic steatosis and nonalcoholic fatty liver disease [69]. Despite the growing body of evidence, a direct causal relationship between OSA and dyslipidemia is yet to be established, and more studies are required.

### 3.7. Increased All-Cause and Cardiovascular Mortality

In the Sleep Heart Health Study, the all-cause mortality was noted to be significantly higher among patients with AHI > = 30/h, especially in 40–70-year-old males [70]. Similarly, a diagnosis of OSA among participants in the Multiethnic Study of Atherosclerosis (MESA) study was associated with a 2.2 higher incidence of CV events and a 2.4 increase in mortality over 7.5 years [71]. The associations are stronger as the severity of OSA increases [72].

## 4. Management

### 4.1. Weight Loss

Obesity is the most consequential risk factor for OSA. It has been estimated that 58% of moderate to severe OSA can be attributed to obesity [73]. A 10% gain in weight increases the odds of developing moderate-to-severe OSA approximately 6-fold and was associated with a 32% increase in AHI. Conversely, a 10% weight loss resulted in a 26% decrease in AHI indicating that weight control may be effective in managing OSA [74].

Weight loss and exercise training interventions improve sleep efficiency and oxygenation but can only lead to full remission of OSA in patients with mild disease [74]. The most remarkable results have been reported with surgical weight loss. Bariatric surgery not only significantly improves AHI and many polysomnographic parameters, but a significant number of patients also have complete OSA remission following surgery [75–77]. Roux-en-Y gastric bypass and sleeve gastrectomy are the most successful bariatric procedures at inducing OSA resolution [75]. Greater weight loss following bariatric surgery is associated with a greater likelihood of OSA remission [75].

### 4.2. Positive Airway Pressure

Continuous positive airway pressure (CPAP) continues to be the primary therapy for patients with OSA due to its impact on both symptoms and quality of life [78, 79]. CPAP targets airway collapsibility by providing a constant level of positive pressure across inspiration and expiration, acting as a mechanical splint for the pharyngeal airway. The prescribed CPAP pressure requires an overnight laboratory titration study to determine the pressure required to eliminate all snoring, apneas, and hypopneas during all sleep stages and in all body positions.

Since CPAP has been shown to improve AHI and correct physiologic disturbances such as hypoxemia, both predictors of CV disease, it has been hypothesized that CPAP treatment may be associated with reduced rates of CV complications. This hypothesis remains unconfirmed.

The Sleep Apnea Cardiovascular Endpoints (SAVE) study randomly assigned over 2700 patients with moderate-to-severe OSA and a history of CV disease to usual care plus CPAP versus usual care alone [80]. While CPAP improved AHI, daytime sleepiness, and health-related quality of life, it did not reduce the number of serious CV events. Similar negative findings were found in the RICCADSA and the ISAACC studies, which investigated CPAP-related CV reduction following PCI and acute coronary syndrome, respectively [81, 82]. Furthermore, a meta-analysis of 9 randomized trials that compared CPAP with no active treatment in adults with OSA and CV disease found that the CPAP did not prolong survival or reduce CV events [83].

Poor adherence to CPAP therapy across these studies complicates the interpretation of these findings. A CPAP usage threshold of ≥4 hour per night is needed for it to maintain its efficacy [84]. The SAVE study had a low average CPAP use of 3.3 hour/night, which raises concern that the results were negative due to poor therapy adherence. Additionally, the subgroup of patients in the study who met the ≥4 hour per night CPAP threshold had a significant reduction in CV events [80]. Subgroup analyses of the RICCADSA and ISAACC studies similarly found that good CPAP adherence led to a significant reduction in CV events [81, 82]. Unfortunately, less than half of patients reach the adherence threshold. Outside the controlled environment of a clinical trial, adherence data may be even more alarming. A review of sleep laboratory records found that approximately 30% of patients prescribed CPAP never actually initiate treatment, and among those patients who do initiate CPAP, a significant proportion abandon it within 1 year [85]. There are 2 ways to setup CPAP for patients—remote and face to face (F2F). With the advent of the COVID-19 pandemic, according to a multicenter study, the F2F setup decreased drastically from 98.9% prepandemic to 1.1% during the pandemic. The study shows a significant drop in CPAP adherence as well [86]. However, one single-center study shows successful implementation of remote diagnostic and treatment pathways for CPAP with similar adherence patterns to the F2F setup pathways [87].

Bilevel positive airway pressure (BPAP) allows different pressures to be used in inspiration and expiration. Therefore, it is potentially capable of treating OSA at a lower mean pressure than CPAP and can help augment ventilation via pressure support. The AHI and sleepiness metric benefits of BPAP are like CPAP. A potential benefit of BPAP over CPAP is the improved comfort due to a lower pressure during exhalation, which may improve patient adherence [88]. BPAP can be considered for patients intolerant of CPAP pressure or who require additional ventilatory support such as in the setting of obesity, chronic obstructive pulmonary disease, or neuromuscular disease.

### 4.3. Oral Appliances

Oral appliances (OAs) and mandibular advancement devices (MADs) can be used as an alternative to CPAP for mild to moderate OSA or in patients who do not tolerate or refuse CPAP [89]. These devices work by lifting the mandible forward and reducing pharyngeal collapsibility [90]. Patient adherence to these devices is greater than with CPAP. Contraindications to use of MADs include severe periodontal disease, severe temporomandibular joint disorders, inadequate dentition, and severe gag reflex [91].

OAs and MADs have been shown to reduce AHI and increase oxygen saturation, but not as effectively as CPAP [4]. A meta-analysis found that among patients with OSA, both CPAP and MADs were associated with reductions in BP and both therapies had statistically similar BP reductions [92]. However, the impact of OAs on resistant hypertension and other CV outcomes has not been evaluated.

### 4.4. Surgical Implanted Stimulation Devices

A hypoglossal nerve stimulator is an implantable pacemaker-like device that delivers electrical pulses to the hypoglossal nerve that are synchronized with ventilation [93]. This protrudes the tongue, opening the pharyngeal airway and maintaining patency. The Stimulation Therapy for Apnea Reduction (STAR) Trial was the landmark trial assessing therapy outcomes of this neurotherapeutic device in the treatment of moderate to severe OSA [93]. After 1-year, the median AHI reduction was 68%, and oxygen desaturation index was similarly reduced by 70% from baseline. This benefit was found to be sustained at 3-year follow-up [94]. A meta-analysis of 12 studies evaluating hypoglossal nerve stimulation corroborated the significant improvements in sleep and quality of life indices with a low long-term complication rate [95]. The impact of hypoglossal nerve stimulation on CV outcomes has not been evaluated.

Hypoglossal nerve stimulation is currently indicated for select patients with an inability to use CPAP, a central apnea index < 25% of the AHI, no complete concentric collapse on drug-induced sleep endoscopy, and a recommended body mass index (BMI) < 35 kg/m^2^ [96]. However, expansion of these criteria is likely to occur as a cohort of patients implanted outside of the traditional criteria had similar reductions in postoperative outcome measures [97].

## 5. Effect of OSA Therapy on the CV Morbidity and Mortality

While there is strong evidence that untreated OSA is associated with increased CV mortality and morbidity, questions have been raised about the benefit of CPAP therapy on CV outcomes.

### 5.1. Hypertension

The effect of CPAP on hypertension has been widely investigated. CPAP use has been found to be accompanied by a modest 2 to 3 mmHg fall in 24-h blood pressure compared with sham or placebo treatment [98–100]. CPAP appeared most effective at lowering blood pressure at night, reversing nondipping of nocturnal blood pressure in OSA patients [101, 102]. Overall, CPAP has a modest and variable effect of blood pressure. Patients with more severe OSA, resistant hypertension, and better CPAP compliance may have more substantial BP reduction with CPAP [54]. A poor blood pressure response to CPAP may indicate that the underlying cause of hypertension is unrelated to OSA. Other pathophysiological mechanisms such as obesity, salt intake, and volume overload are unaffected by CPAP. It is important to note that in patients with moderate-severe OSA and obesity, CPAP use combined with weight loss has incremental benefits in terms of insulin resistance, blood pressure, and triglyceride levels [103].

### 5.2. Heart Failure

CPAP trials in HF have evaluated the impact of treating OSA on surrogate CV end points rather than hospitalization or mortality. CPAP treatment in HF patients helps minimize the sympathetic hyperactivation imposed by OSA as it has been shown to decrease vascular and myocardial sympathetic nerve function and improves myocardial energetics [101, 102]. In a randomized trial involving 24 moderate-severe OSA patients with HF with reduced ejection fraction, 30 days of CPAP treatment led to an improvement in left ventricular systolic function, significantly increasing the echocardiographic assessed ejection fraction by 9% [104]. A sham-controlled trial of newly diagnosed OSA patients with left ventricular diastolic abnormalities found that 12-weeks of CPAP treatment could improve diastolic function [105].

Observational data of Medicare beneficiaries suggest a lower mortality rate, decreased readmission, and lower health care costs in HF patients with CPAP-treated OSA compared with untreated OSA [106]. In a French nationwide real-world study, patients with OSA with continued CPAP use were compared with patients that discontinued CPAP therapy. The latter had a significantly higher mortality rate which could be related to higher incident heart failure among these patients [107]. These results are in contradiction with RCTs that that suggest no reduction in cardiovascular events with the use of PAP therapy when compared with usual care [80–82].

### 5.3. Arrhythmias

The effects of CPAP treatment on cardiac arrhythmias have not been thoroughly studied. Observational data suggest that OSA patients treated with CPAP have significantly reduced occurrence of paroxysmal AF compared with untreated patients [108]. Likewise, CPAP treatment is associated with a significantly decreased rate of atrial fibrillation recurrence after electrical cardioversion or ablative therapies [109, 110]. However, a recently conducted randomized trial of 579 patients with paroxysmal atrial fibrillation and moderate to severe OSA failed to show a statistically significant reduction in the AF burden with CPAP treatment [111].

### 5.4. Coronary Artery Disease

As mentioned previously, in the SAVE trial CPAP failed to show a significant reduction in CV events in patient with OSA and established CV disease [80]. However, benefit may be present in patients highly adherent to CPAP therapy. Additionally, patients with severe OSA and at highest risk of CV events were excluded from the trial. Therefore, the debate on whether CPAP therapy decreases the risk from coronary artery disease remains unsettled and controversial.

### 5.5. Sleep Apnea and the COVID-19 Pandemic

Sleep apnea has been shown to be a risk factor for severe COVID-19 pneumonia requiring hospitalization and respiratory failure even [112, 113]. Some studies focus on association between sleep apnea and increased COVID-19 related mortality [114]. It should be emphasized however that the COVID-19 pandemic also had a detrimental effect on overall management of sleep apnea.

The pandemic has affected overall outpatient care for patients. Data from 2020 shows that number of in-person outpatient visits had initially declined by about 60% [115]. However, in compensation, the telemedicine appointments rapidly rose [116]. By the end of the year, the overall outpatient visits among adults remained stable as compared to the prepandemic figures. This trend was noticed across all specialties with minor variations among them. Data focused on sleep clinic appointments is scarce [115]. However, survey-based studies suggest drastic decline in the number of in-laboratory PSG and a push towards adopting remote technologies like home sleep apnea testing. The step most risky for healthcare professionals is PAP titration because of generation of aerosols and increased transmissibility of the virus. Use of autotitrating CPAP becomes especially handy in this situation [117–119]. PAP therapy is most widely used treatment strategy for sleep apnea. The pandemic has caused global supply-chain disruptions, mainly because of restriction of movement of goods across borders and the resulting increase in cost of delivery [120]. This has certainly affected timely delivery of CPAP equipment including PAP filters. To make this worse, the US FDA had to recall some Philips CPAP equipment because of a potential health hazard [121]. The physician community has been appropriately adaptive evidenced by recommendations issued by institutions (like Centers for Disease Control, American Academy of Sleep Medicine, and Australian Sleep Association) regarding reopening of sleep medicine practices. Some studies assessed the adherence patterns of CPAP therapy among patients who already had a diagnosis of sleep apnea. Interestingly, they noted an overall improvement across all age groups. They contemplate that it could be the fear of becoming ill driving this behavior [122].

## 6. Conclusion

OSA is highly prevalent and still underdiagnosed especially among patients with CV diseases. CV mortality and morbidity are increased in the presence of OSA as it is associated with an increased risk of resistant hypertension, HF, arrhythmias, and coronary artery disease. The use of screening questionnaires should be routine, but a formal sleep study is fundamental in establishing and classifying OSA. Laboratory PSG is the gold standard but still significantly adds to the logistical barriers in management of OSA. HSAT is a reasonable alternative but is not suitable for many patients with cardiovascular diseases like heart failure. Positive airway pressure remains the mainstay of treatment but unfortunately is associated with poor adherence patterns. Even though other options like oral appliances and stimulation devices are available in the market, they lack evidence demonstrating reduction in CV morbidity and mortality. Further study is required to fully elucidate the favorable effects of OSA treatment modalities on CV diseases.

## Figures and Tables

**Box 1:**
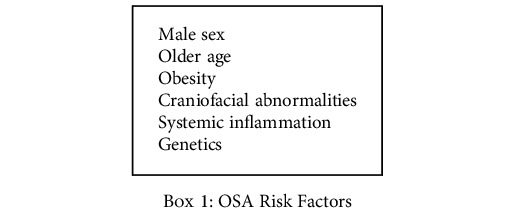
OSA Risk Factors
